# Urban eddy covariance measurements reveal significant missing NO_x_ emissions in Central Europe

**DOI:** 10.1038/s41598-017-02699-9

**Published:** 2017-05-30

**Authors:** T. Karl, M. Graus, M. Striednig, C. Lamprecht, A. Hammerle, G. Wohlfahrt, A. Held, L. von der Heyden, M. J. Deventer, A. Krismer, C. Haun, R. Feichter, J. Lee

**Affiliations:** 1 Institute of Atmospheric and Cryospheric Sciences, University of Innsbruck, Innsbruck, Austria; 2 Institute of Ecology, University of Innsbruck, Innsbruck, Austria; 3 Atmospheric Chemistry, University of Bayreuth, Innsbruck, Germany; 4 Department of Geography, University of California, Berkeley, USA; 5 Abteilung Waldschutz, Amt der Tiroler Landesregierung, Innsbruck, Austria; 6 Abteilung Geoinformation, Amt der Tiroler Landesregierung, Innsbruck, Austria; 7 Amt für Verkehrsplanung, Umwelt, Magistrat III Stadt Innsbruck, Innsbruck, Austria; 8 National Centre for Atmospheric Science and Department of Chemistry, University of York, York, UK

## Abstract

Nitrogen oxide (NO_x_) pollution is emerging as a primary environmental concern across Europe. While some large European metropolitan areas are already in breach of EU safety limits for NO_2_, this phenomenon does not seem to be only restricted to large industrialized areas anymore. Many smaller scale populated agglomerations including their surrounding rural areas are seeing frequent NO_2_ concentration violations. The question of a quantitative understanding of different NO_x_ emission sources is therefore of immanent relevance for climate and air chemistry models as well as air pollution management and health. Here we report simultaneous eddy covariance flux measurements of NO_x_, CO_2_, CO and non methane volatile organic compound tracers in a city that might be considered representative for Central Europe and the greater Alpine region. Our data show that NO_x_ fluxes are largely at variance with modelled emission projections, suggesting an appreciable underestimation of the traffic related atmospheric NO_x_ input in Europe, comparable to the weekend-weekday effect, which locally changes ozone production rates by 40%.

## Introduction

The nitrogen cycle^1^ is essential for maintaining the oxidizing capacity of the atmosphere and regulating ozone in the lower atmosphere^2^. Perturbations due to rapid industrialization and agricultural activities have led to a significant increase of atmospheric nitrogen oxides (NO_x_) during the 20^th^ century^3^. A regionally intense buildup of photochemical smog due to the presence of nitrogen oxides, CO and non-methane volatile organic compounds (NMVOC) was first identified in the US and attributed as the main cause of severe ozone pollution in many areas^4^. Decades of subsequent research activities ranging from detailed laboratory^5, 6^ and smog chamber^7–9^ studies to large scale field campaigns^10–12^ have led to a reasonably good mechanistic understanding of the formation of tropospheric ozone, which is characterized by a complex nonlinear relationship between NO_x_ and reactive carbon species^13^. This interdependency gives regulators two key strategies to mitigate ozone pollution. The effectiveness to control ozone thereby very much depends on the ratio between ambient OH reactivity and NO_x_ concentrations^14^, which can be described by relatively simple analytical relationships^15^. The development of mechanistic regional^16^ and global air chemistry models^17^ has further given regulators and scientists powerful tools to study tropospheric ozone formation^16^, where the mitigation of NO_x_ emissions has emerged as one of the key air pollution control strategies for ozone^18–20^ and more recently also for particulate matter with a diameter of 1 μm or less (PM1)^21^. Due to the toxicity, nitrogen dioxide (NO_2_) is also regulated as a hazardous air pollutant itself^22^. For example, in Europe regulatory action under the EU Thematic Strategy on Air Pollution is in place to limit urban street canyon NO_2_ concentrations to 40 μg/m^3^ per year (or 200 μg/m^3^/h on less than 18 days/year)^23^. Current trends across European air quality networks show that regulatory thresholds of NO_2_ are violated at many stations, which does not seem to be limited to large population centers anymore (ref. 24, *SI*). In fact many rural areas and smaller towns see NO_2_ concentration levels rivaling those of large metropolitan areas. Owing to the spatiotemporal variability and uncertainty of different anthropogenic NO_x_ sources, it is difficult to attribute emission uncertainties to specific sectors in complex bottom-up emission inventories^25^ or top-down remote sensing assessments^26^. Recently evidence has accumulated that rapid shifts in transportation fuels can have significant impacts on air quality^27, 28^. In Europe for example the question about the increasing penetration of Diesel cars raises concerns as to what extent such a technological change has been counterproductive to mitigating atmospheric NO_2_ pollution under new emission regulation standards^19, 29^. The United States environmental protection agency’s (US EPA) notice of violation of the Clean Air Act to a German automaker regarding Diesel engines has sparked a number of new real world driving (RDE) emission tests across Europe, which show significant manufacturer and vehicle specific variability^30, 31^. These new data suggest that the impact on up-scaled average fleet emissions needed for accurate air quality predictions remains unclear^32^.

A number of urban flux measurement sites for energy and CO_2_ have been established highlighting their potential for surface-atmosphere exchange studies^33, 34^. In contrast, similar measurements for reactive gases are often still quite limited, owing to the complexity of the required measurement systems. A set of recently conducted urban ground based and airborne NMVOC flux measurements revealed the usefulness to test bottom-up emission inventories and revealed significant discrepancies for some species^35–39^. Urban flux measurements for NO_x_ are even more scarce^32, 40, 41^ indicating that constraints on emission sources in urban areas can be quite uncertain. Here we improve upon existing work, by simultaneously measuring NO_x_, selected tracer NMVOCs, CO and CO_2_ leading to a well constrained flux dataset, that allows testing our understanding of prominent NO_x_ emission sources.

## Results

### Obtaining ensemble average statistics on fleet emissions by eddy covariance flux measurements

**Figure 1:**
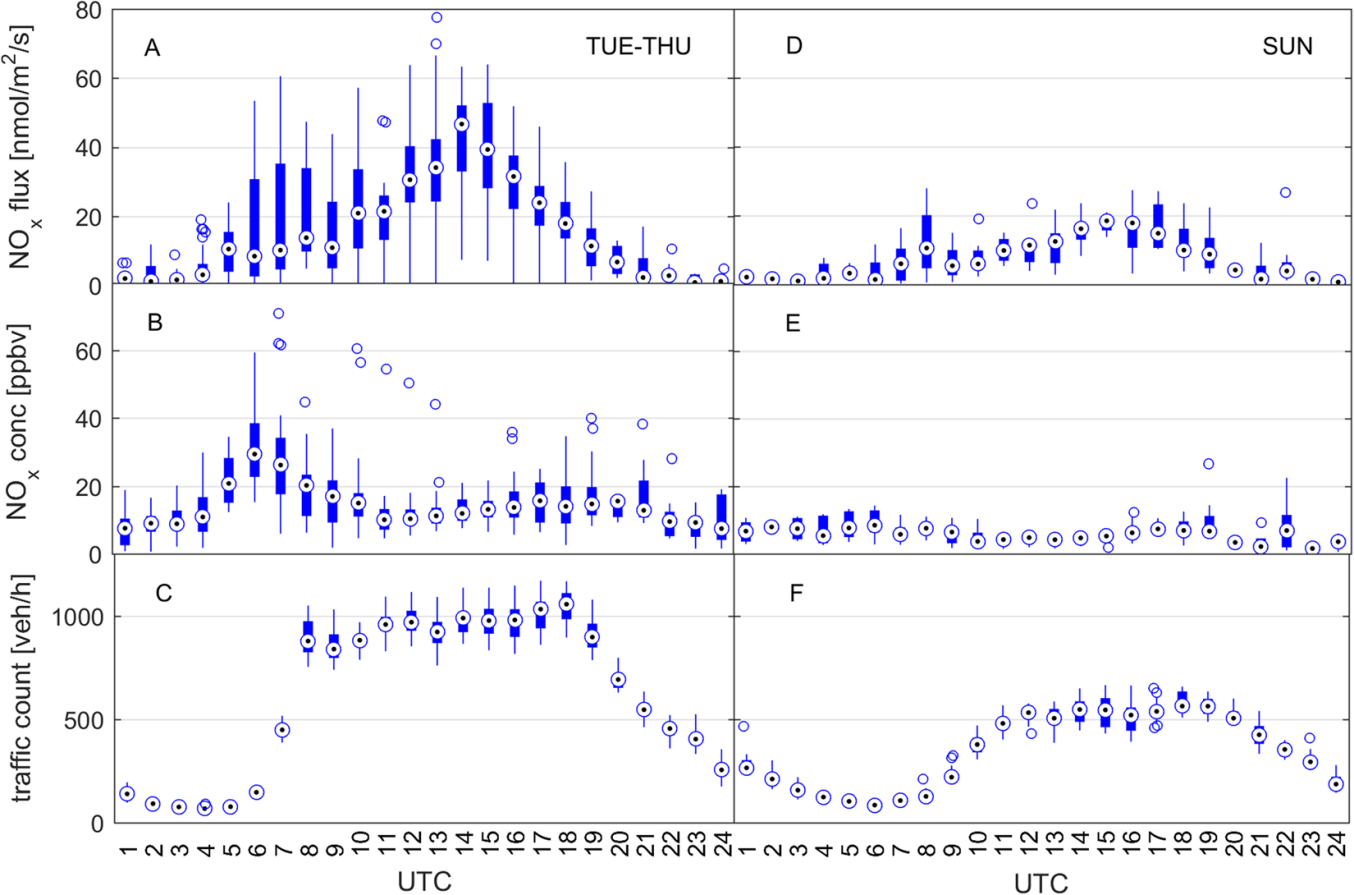
Statistical plot of measured NO_x_ fluxes, mixing ratios and traffic count data. The center dot shows the ensemble median, where the box around it represents one standard deviation and whiskers the 25 and 75% percentile. Individual extreme values are plotted as open circles. Panels A, B and C represent weekdays (i.e. TUE-THU; composite of 609 individual data points) and panels D, E and F depict Sundays (193 individual data points).

A comprehensive set of eddy covariance measurements for NO_x_, marker NMVOC, CO and CO_2_ at an urban location allows a direct comparison of relative flux ratios with bottom-up emission sources. The study site located in Innsbruck (N 47°15′51.50″, E 11°23′6.77″) at the center of the Inn valley, represents one of the most strategically important Alpine crossing points for the transport of goods between Northern and Southern Europe. Each year approximately 6 million vehicles^42^ pass through the east-west facing valley, which is about 10 km wide surrounded by mountain ridges about 2.5 km high. The valley topography leads to a very predictable and pronounced wind system characterized by a topographic amplification factor (TAF) of about 3^43^. Due to the combination of significant traffic induced NO_x_ emissions and increasingly stringent NO_2_ limit values, the area is in non-attainment. Local authorities are facing legal proceedings by the European Commission for their failure to control excessive levels of nitrogen oxides (Fig. S2), similar to many areas across Europe^23^. Tracer flux relationships allow investigating to what extent urban emissions are caused by (a) traffic, (b) urban residential and (c) biomass burning/biofuel activities. Figure 1 shows the diurnal evolution of Weekday (Tuesday-Thursday) and Sunday NO_x_ fluxes and concentrations along with mean traffic count data at the site during the measurement campaign (July – October, 2015). Median measured midday NO_x_ mixing ratios in Innsbruck are comparable to values reported for central London (10–14 ppbv), while corresponding observed fluxes are about a factor of 3–4 lower (i.e. 3000–4000 ng/m^2^/s vs 700–1440 ng/m^2^/s)^32^. These observations are consistent with the idea of an intensification of air pollution proportional to TAF, and a corresponding effective lower air volume that pollutants are being mixed into in steep valleys (Fig. S2, ref. 43). This comparison likely indicates that a much stronger reduction of NO_x_ emissions from the transport sector would be required in the Alps than for example in London in order to achieve current air pollution standards along one of the busiest EU transport corridors across the Alps. Several lines of evidence exclude significant presence of biomass burning during the present study. The average ratio between benzene and toluene fluxes exhibited a typical value (3.3 ± 0.7; R^2^ = 0.85) characteristic for urban emission sources, dominated by fossil fuel combustion and evaporative/cold-start emissions. The correlation between acetonitrile and benzene, toluene, NO_x_ or CO_2_ fluxes was low with an R^2^ of 0.07, 0.08, 0.02 and 0.06 respectively. We also did not observe significant excursions of other species recently suggested as additional biomass burning markers^44^ such as furfural and furan showing a correlation coefficient of R^2^ < 0.2 above their background fluxes. We observed an excellent correlation between CO_2_, benzene and NO_x_ fluxes (CO_2_/NO_x_: R^2^ = 0.86; benzene/NO_x_: R^2^ = 0.75). The covariance between between NO_x_ and CO_2_ (benzene) fluxes yielded values of 0.91 (0.86). We interpret these observations such that benzene, NO_x_ and CO_2_ emissions are dominated by road traffic with contributions from residential combustion sources.

### Benchmarking urban source emission ratios and inventories

**Figure 2:**
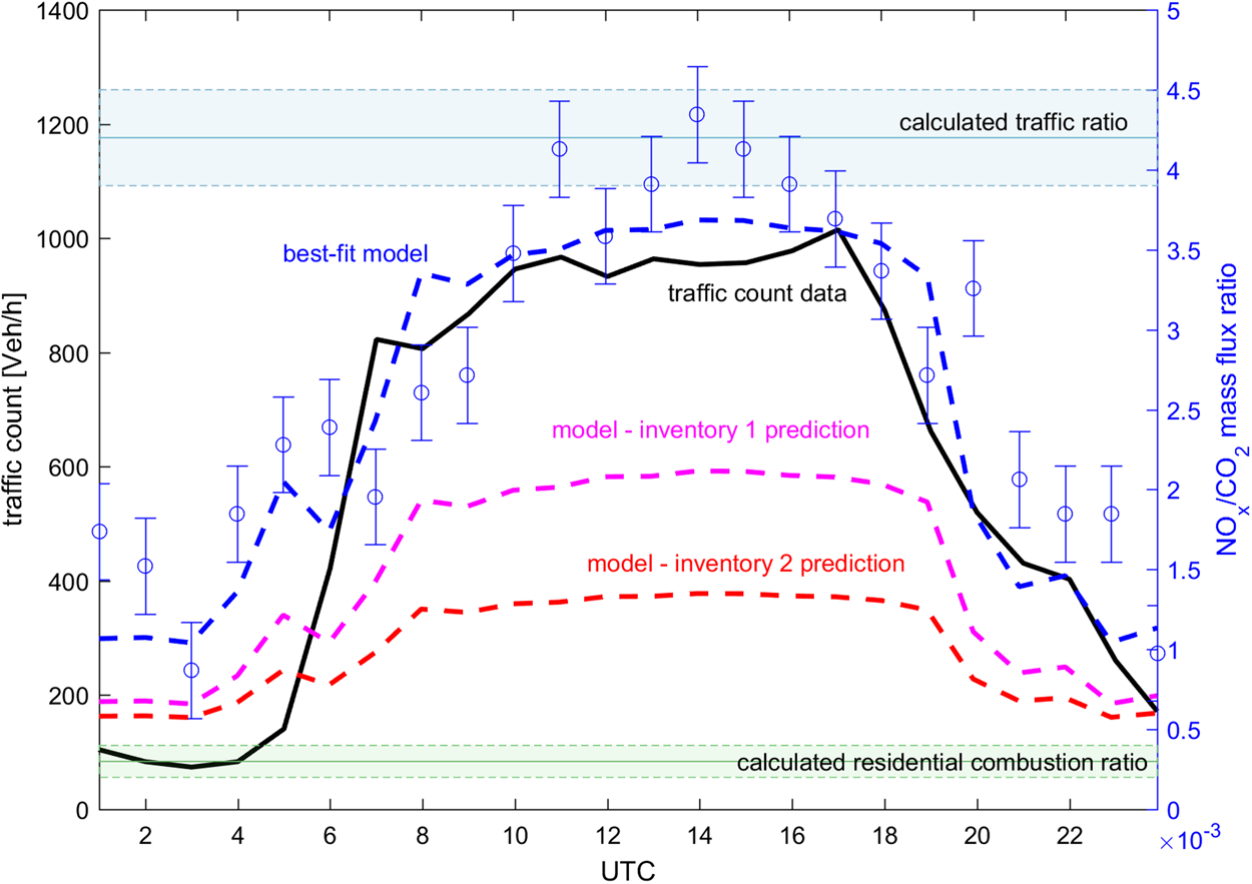
Diurnal cycle of median traffic count data (black line - left axis), measured (blue circles) and model fitted (dashed blue line) mass flux ratio of NO_x_/CO_2_ (right axis). The corresponding calculated end members for traffic and residential combustion ratios are indicated by blue and green horizontal lines. The shading reflects one standard deviation. In addition colored dashed lines show predictions using fixed emission ratios from COPERT (magenta) and ACCMIP (red).

To gain a more quantitative insight, we investigated flux ratios between NO_x_ and CO_2_ (F_NOx_/F_CO2_; Fig. 2). The advantage of this approach is that it allows determining the actual ensemble average of different emission sources based on measured flux ratios, similar to an end-member un-mixing regression analysis. This allows us to compare our measurements to relative emission strengths reported in emission models and inventories. For large scale emission inventories (e.g. grid cells > 1 km^2^) this approach also circumvents uncertainties related to assumptions of various downscaling approaches. The observed F_NOx_/F_CO2_ ratios follow a diurnal cycle showing a ~40–50% variation throughout a day, which reflects the pronounced fluctuation of traffic activity across the city (e.g. ranging from about 97 vehicles/h at night to 890 vehicles/h during daytime at a traffic count station within the flux footprint). Since we can exclude significant industrial emissions within the flux footprint as well as biomass burning, the variation of F_NOx_/F_CO2_ should exhibit the characteristic behavior of city scale sources comprised of (1) a combination of vehicular emissions and (2) residential/domestic combustion sources (e.g. oil and gas heating units). A minimization routine (SI) allowed un-mixing these two end-members of the compositional data, reflecting the actual emission ratios for traffic and urban residential combustion sources (Fig. 2). The fitted model (SI) can reproduce the diurnal cycle and activity factors reasonably well, leading to a NO_x_/CO_2_ emission ratio for traffic of 4.2(±0.3) × 10^−3^ ([mg/m^2^/h] NO_x_/[mg/m^2^/h] CO_2_), and 0.20(±0.05) × 10^−3^ ([mg/m^2^/h] NO_x_/[mg/m^2^/h] CO_2_) for residential combustion sources. These calculated ratios are also depicted in Fig. 2 by horizontal shaded blue and green lines. The activity factors suggest that the ratio is dominated by traffic, comprising about 85% of the activity averaged over the entire day (and >95% during peak traffic). We also investigated NO_x_/benzene flux source ratios revealing comparable differences as observed for F_NOx_/F_CO2_. The CO_2_ flux weekend-weekday effect and CO/CO_2_ ratios close to a recent road tunnel study^45^, all imply that a biogenic influence on CO_2_ fluxes due to photosynthetic uptake or respiration can be considered negligible at this site. The obtained NO_x_/CO_2_ flux ratio for traffic is significantly larger than predicted by a number of state of the art emission inventories and emission standards (Table 1). Generally, we observe 50–70% higher NO_x_ emissions relative to CO_2_ from road traffic than what is calculated with the most recent traffic emission models. In these detailed bottom – up models, mobile source emissions are treated for different engine sizes and fuels, that, in the past, relied on standardized protocols obtained in test facilities, but were recently updated based on a number of RDE tests^30, 31^. Exhaust from modern gasoline powered engines, despite higher ignition temperatures than those powered by Diesel, can be effectively treated for NO along with NMVOC (and CO) using a three way catalytic (TWC) converter. TWC treatment can lead to a 10 fold reduction of NO. This has been hard to achieve for Diesel powered cars, which nowadays mostly rely on selective catalytic reduction due to the high air to fuel ratio during combustion. Generally, different combustion and exhaust treatment characteristics result in significantly higher NO_x_/CO_2_ emission ratios for Diesel powered cars than for gasoline. The modelled fleet average contribution suggests that at least 90% of urban NOx emissions should originate from Diesel driven vehicles at the present location. 85% is modelled to be emitted by the passenger car fleet based on the COPERT model and TRACCS database (ref. 46, *SI*). The current Austrian passenger car fleet comprises about 50% Diesel cars and the percentage across Europe grew at a substantially faster rate compared to the US^47^. Based on our measurements the current average Austrian car fleet emits about 36 times more NO_x_ per CO_2_ molecule compared to the US TIER II emission standard and a factor of 8–10 more than Euro 6 emission standards^31^. Factoring in differences in fuel economy between the European and US car fleet, this would equate to about an order of magnitude more NO_x_ emissions per travelled distance compared to newly introduced emission standards. How comparable are these results to other European countries? Diesel engines dominate the European passenger car market: 55% of all newly registered vehicles in the EU were powered by Diesel-fuel in 2012^42^; the penetration of Diesel cars of the two largest European economies bordering the Alps ranges from 30% (Germany) to 70% (France)^42^, more than an order of magnitude higher than in the US^47^. When comparing measured NO_x_/CO_2_ flux ratios with current inventories used for IPCC atmospheric chemistry/climate^48^ and air quality models^49^ we obtain a discrepancy up to about a factor of 3–4 (Table 1). Incidentally, a recent comprehensive model evaluation has suggested significant discrepancies between regionally modelled and observed surface NO_x_ concentrations that seemed worse (e.g. by a factor of 2 during summers) over Europe than over the US^25^. While a number of uncertainties (agricultural emissions, vertical mixing, biomass burning emissions, deposition) can potentially result in modelled concentration biases in these models, our measurements suggest that road transport related biases likely contribute significantly to these discrepancies as they account for about half of the total NO_x_ emissions across Europe^50^.

**Table 1:** Measurement – inventory comparison (i.e. measured/modelled flux (emission) ratio). The uncertainty range is given by sub- and superscripts. PC: passenger cars; LCV: light commercial vehicle.

Measurement/inventory ratio or Measurement/emission standard ratio		NO_x_/CO_2_ NO_x_/CO	NO_x_/benzene
INNAQS/COPERT^1,#^		${1.7}_{1.6}^{1.8}$	${3.9}_{3.1}^{4.7}$
INNAQS/HBFA3.2^2^		${1.5}_{1.4}^{1.6}$	N/A
INNAQS/ACCMIP (traffic)^3^		${4.0}_{2.8}^{5.3}$*	${2.1}_{1.7}^{2.5}$
INNAQS/EMEP (traffic)^#^		${2.9}_{2.6}^{3.3}$*	N/A
INNAQS/US Tier II^4^		${36.0}_{33.4}^{38.6}$	N/A
		NO_x_/CO_2_ PC and LCV < 1305 kg	NO_x_/CO_2_ LCV 1305 kg–3500 kg
INNAQS/Euro6^5^	Diesel	${4.7}_{4.3}^{5.0}$	${4.5}_{4.1}^{4.8}$
	Petrol	${12.6}_{11.7}^{13.5}$	${19.5}_{18.1}^{20.9}$
INNAQS/Euro5^5^	Diesel	${2.1}_{1.9}^{2.3}$	${2.0}_{1.8}^{2.1}$
	Petrol	${12.6}_{11.7}^{13.5}$	${19.5}_{18.1}^{20.9}$
INNAQS/Euro4^5^	Diesel	${1.5}_{1.4}^{1.6}$	${1.4}_{1.3}^{1.5}$
	Petrol	${4.7}_{4.4}^{5.0}$	${14.5}_{13.5}^{15.5}$
INNAQS/Euro3^5^	Diesel	${0.75}_{0.7}^{0.8}$	${0.9}_{0.85}^{1.0}$
	Petrol	${2.5}_{2.3}^{2.7}$	${18.0}_{16.7}^{19.3}$

^1^COPERT emission model (*SI*).

^2^HBFA 3.2 (*SI*). Comparison with HBFA 3.3, that was published during the copy editing phase, yielded an average bias of 1.2.

^3^ACCMIP – (*SI*).

^4^US EPA^22^.

^5^EEA^24^.

*For inventories that do not explicitly report CO_2_, we converted data using the measured midday range of CO to CO_2_ flux ratios (3.6 to 4.6 ppbv/ppmv), which fall close to a recent evaluation based on a road tunnel study^45^.

^#^Data are trend adjusted for 2015 according to GAINS (http://gains.iiasa.ac.at/models/)^23^.

### What is the impact on atmospheric chemistry?

The weekend – weekday effect (Fig. 1) allows to gain insight into changing NO_x_ and NMVOC fluxes on ozone production in more detail. In Austria, heavy duty vehicle traffic (trucks heavier than 7.5t and all road trains) is banned between Saturday 15:00 and Sunday 22:00 and on public holidays between midnight and 22:00. Traffic count data generally show a pronounced difference in driving habits resulting in a factor of 1.9 ± 0.2 lower vehicle counts on Sunday than on weekdays. We calculated typical ozone production rates for midday-afternoon conditions (11–16 h LT), when photochemistry peaks. The corresponding NO_x_ fluxes are a factor of 2.1 ± 0.2 lower on Sundays, closely matching observations of vehicle activity. Benzene and toluene fluxes, representing the variation of anthropogenic NMVOC emissions, were lower by a factor of 1.8 ± 0.3 and 2.0 ± 0.3 respectively. CO_2_ fluxes changed by a similar factor of 2.3 ± 0.5. The weekend-weekday comparison provides an independent confirmation that these pollutant emissions are dominated by traffic activity during the day. Average NO_x_ concentrations are a factor of 2.5 ± 0.2 lower during Sundays. Incidentally, the observed weekend – weekday reduction is comparable to the observed measurement-inventory discrepancies or the effect if an entire car fleet was converted from a Euro 5 (0.18 g/km) to a Euro 6 (0.08 g/km) NO_x_ emission standard. Our measurements therefore allows us to benchmark such a hypothetical regulatory action in the real atmosphere. Changes in local ozone production are calculated following procedures outlined before^15, 51^, where the sensitivity of local ozone production can be approximated by the ratio of radical termination (L_N_) processes (e.g. NO_2_ + OH) and photochemical radical production (Q):

1 $$\frac{{L}_{N}}{Q}\approx \frac{2\delta {O}_{x}-2\delta N{O}_{x}-\delta J}{\delta {O}_{x}+\delta NMVOC-3\delta N{O}_{x}}$$

Here the δ symbol indicates the relative change between weekday and Sunday, J represents the photolysis rates, and O_x_ = O_3_ + NO_2_. All terms on the right side can be inferred from measurements of the weekend effect, where the anthropogenic change of NMVOCs is assumed to follow benzene. There is evidence of a non-neglegible biogenic NMVOC (BVOC) presence at the site (e.g. 20–50% of the NMVOC reactivity) and these BVOCs do not exhibit any anthropogenically related variation between weekdays and weekend. We apportioned the change of NMVOC reactivity therefore into a biogenic and anthropogenic part using data from the PTR-QiTOF-MS instrument. To achieve this, we estimated the total anthropogenic NMVOC reactivity from known urban concentration ratios^52, 53^ scaled to benzene and compared this to the measured reactivity of BVOC, in particular the reactive biogenic marker species isoprene and monoterpenes. This allowed to obtain upper (δNMVOC = δbenzene) and lower (δNMVOC = δbenzene × [reactivity anthropogenic NMVOC]/[reactivity BVOC]) bounds for L_N_/Q. It can further be shown^15^ that the sensitivity of local ozone production can be related to L_N_/Q according to:

2a $$\frac{d\,\mathrm{ln}\,P({O}_{3})}{d\,\mathrm{ln}(N{O}_{x})}=\frac{1-\frac{3}{2}\,\frac{{L}_{N}}{Q}}{1-\frac{1}{2}\frac{{L}_{N}}{Q}}$$

2b $$\frac{d\,\mathrm{ln}\,P({O}_{3})}{d\,\mathrm{ln}(NMVOC)}=\frac{\frac{1}{2}\,\frac{{L}_{N}}{Q}}{1-\frac{1}{2}\frac{{L}_{N}}{Q}}$$

**Figure 3:**
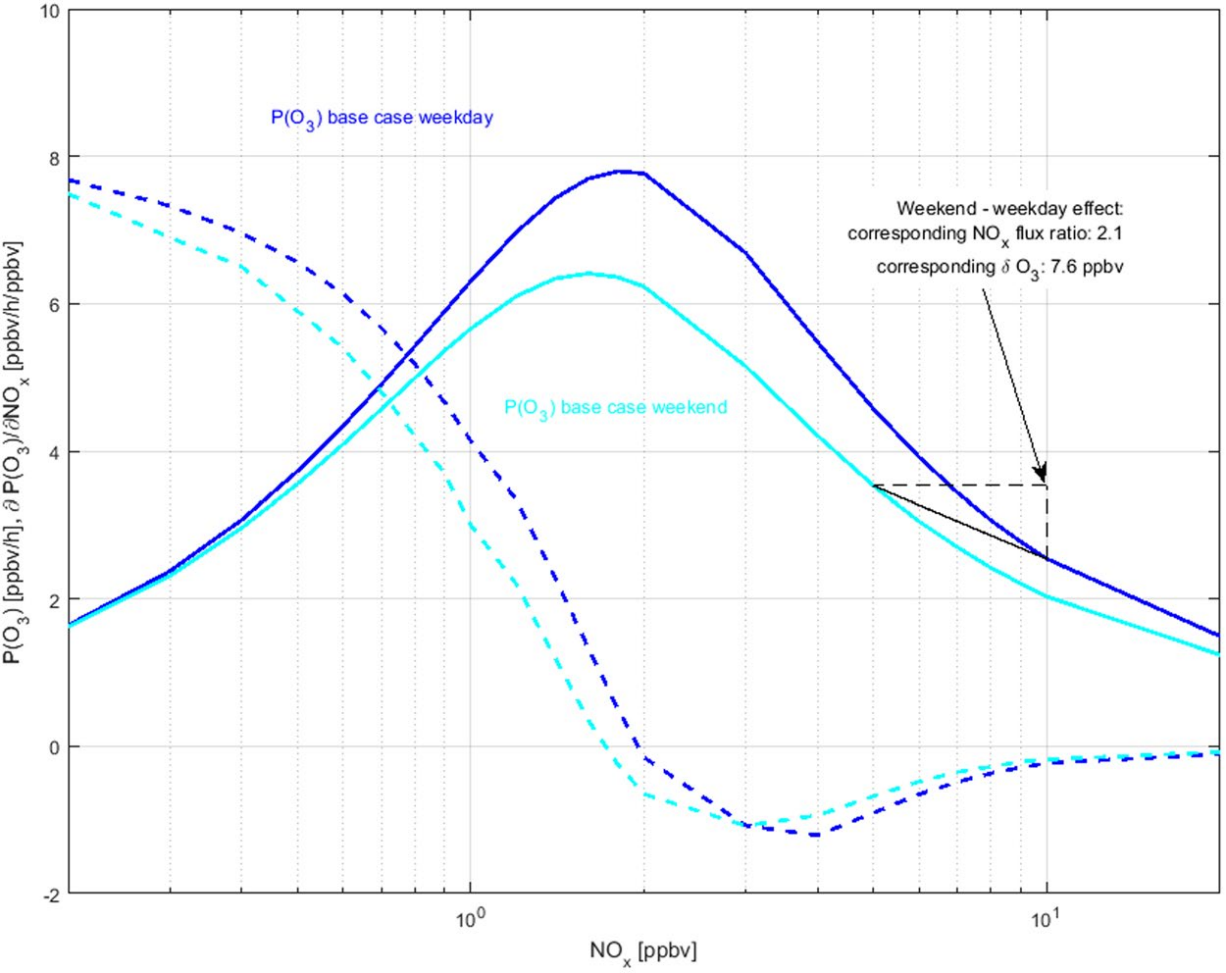
Calculated ozone production rates (solid lines) and derivatives (dashed lines) as a function of NO_x_. Blue and cyan represent base cases for the study site, taking into account changes in O_3_, NO_x_ and NMVOCs concentrations. The solid black line follows the observed weekend-weekday trajectory.

**Figure 4:**
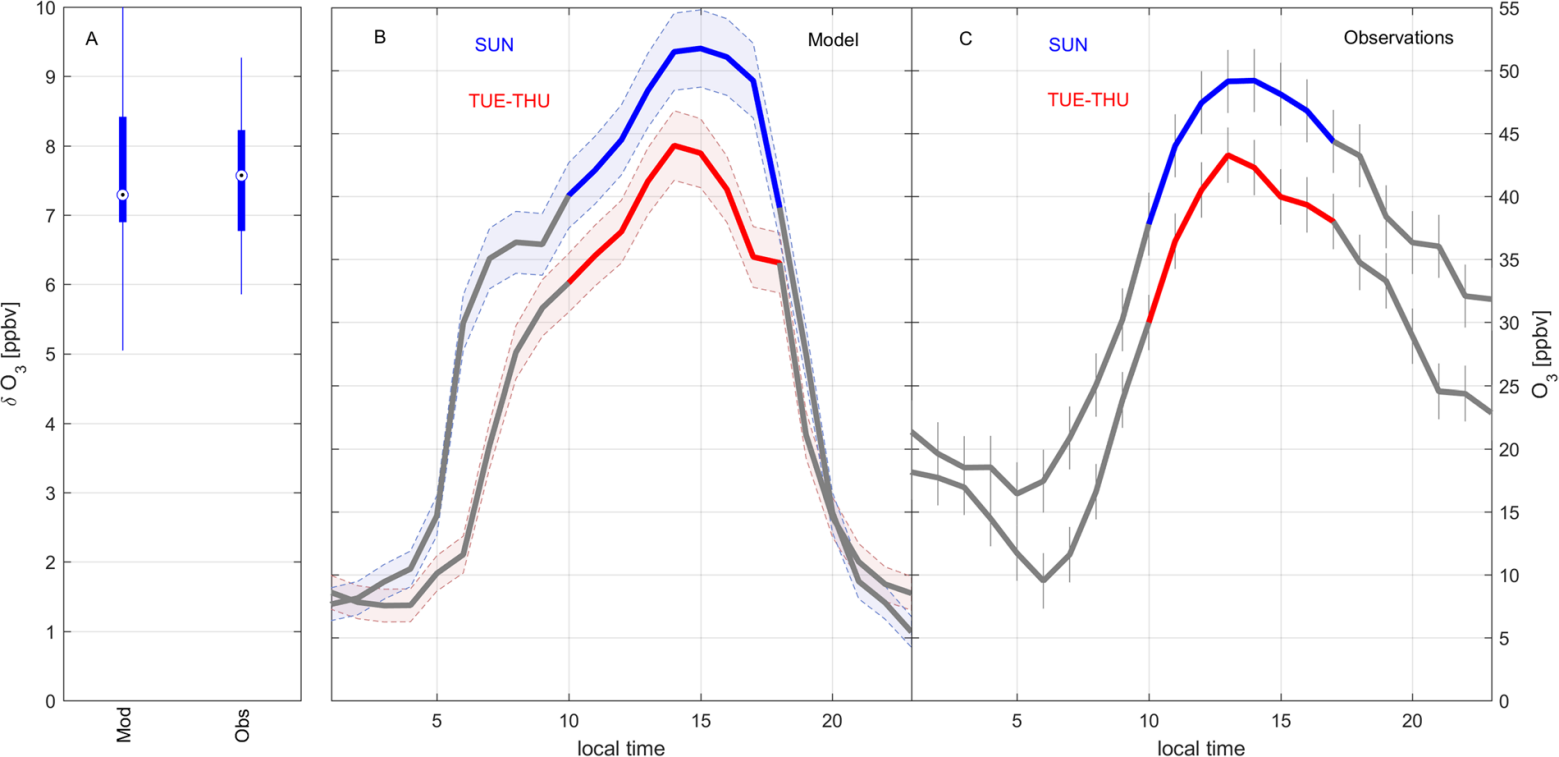
Observed and modelled changes in ozone concentrations on weekdays (Tuesday – Thursday) and weekends (Sunday). Panel A depicts a statistical boxplot showing weekend-weekday differences during daytime, which are represented by the colored sections in panels B and C.

The calculated range for L_N_/Q delimits a ratio between 0.82 and 0.92 (theoretical maximum = 1) and places the site in a NO_x_ inhibited chemical regime for ozone production during weekdays^15, 51^. Incidentally these afternoon values are systematically higher than reported for a rural site in Northern Italy with comparable NO_x_ concentrations, and more closely follow conditions reported for Milan in 1998^54^. Our flux data clearly demonstrate that the weekend effect of ozone production found in Innsbruck is largely a direct result of lower weekend NO_x_ emissions. As a consequence of the dominating nitrogen chemistry for radical loss calculated via eqs 1 and 2, local ozone production will therefore increase by 39% to 70% proportional to a reduction of NO_x_ mixing ratios in Innsbruck. It will decrease between 70% to 85% proportional to a reduction of NMVOC or CO. The impact on gross ozone production (P(O_3_)) was also investigated independently using the Leeds Master Chemical Mechanism (MCM) as a photochemical box model (refs 13 and 55, *SI*, Fig. 3). The model simulates an increase of P(O_3_) by 40% (from 2.5 to 3.5 ppbv/h) on Sundays due to a 2 fold reduction of NO_x_, giving an explanation for the observed weekend effect, which results in an increase of local afternoon ozone concentrations up to 24% (Fig. 4). The model simulation suggests a maximum increase of P(O_3_) up to 3 times (2.5 ppbv/h to 7.8 ppbv/h), if solely NO_x_ concentrations were decreased from current levels to about 2 ppbv (Fig. 3). Below 2–4 ppbv of NO_x_, ozone production would enter the NO_x_ sensitive regime and gradually lead to decreasing P(O_3_). Next, we setup the MCM as a diurnally constrained 0-dimensional diluting box model (*SI*) to study the sensitivity with respect to changes in modelled ozone concentrations between weekdays and weekends. The model is thereby fully constrained by methane, CO, measured NMVOC, NO, photolysis rates, and PBL height along with ancilliary meteorological variables (e.g. temperature, humidity). NO_2_ and ozone are initialized by their measured initial concentrations, but are then allowed to freely adjust during the model run. We chose a spin-up time of 3 days and used model results from the last day of simulation to compare with measured ozone concentrations. Figure 4 shows the diurnal patterns of modelled and observed ozone concentrations. Observed (modelled) peak ozone concentrations are 49 ± 2 (52) ﻿ppbv and 43 ± 2 (44) ppbv on weekends and weekdays respectively. Modelled and observed weekend-weekday differences averaged over daytime hours correspond to 7.2  ±  1 and 7.6 ± 1.2. While the model reproduces the general diurnal ozone cycle and midday peak reasonably well, it underestimates absolute nighttime concentrations exhibiting a stronger diurnal cycle. We attribute this shortcoming mainly to poor knowledge and constraints on entrainment and advection processes that dominate observed nighttime distributions of ozone. While this approach has its limitations, we believe it does a sufficient job for investigating relative daytime changes of ozone concentrations caused by the weekend effect. On average, daytime weekday concentrations are about 7–8 ppbv lower than on weekends. These results can be compared to two quite contrasting areas. In Mexico City^51^, ozone concentrations change very little despite similar relative variations of NO_x_ mixing ratios between weekday and weekend, which can be attributed to a completely NO_x_ saturated environment. On the other hand, a quite pronounced variation is found in the southern California Air basin^56^, where average ozone concentrations are observed to change almost twice as much compared to the present study. We interpret these observations such that they reflect the change in ozone production efficiency (Fig. 4), when going from environments with NO_x_ saturated conditions to more VOC limited conditions. In this context a number of explanations of the weekend effect on ozone mixing ratios are often discussed^51^: (1) different timing of NO_x_ emissions on weekends and associated chemical repartitioning, (2) carryover of previous day pollutants at the surface and aloft, (3) higher weekend VOC emissions, (4) higher weekend photolysis frequencies due to lower aerosol loadings, (5) changes in biogenic emissions due to a different radiation regime caused by lower aerosol loadings and (6) lower overall weekend NO_x_ emissions. We show for the first time, that, for Innsbruck, at least 85% of the NO_x_ concentration change, leading to a 7–8 ppbv ozone increase on weekends, can be directly associated with a change of the overall local atmospheric NO_x_ flux.

## Discussion

Continued urbanization in conjunction with rapid technological changes in the mobility sector poses a challenge for accurate up to date predictions of pollutant emissions. By constraining the actual fluxes of nitrogen oxides, NMVOC, CO_2_ and CO into the atmosphere our measurements provide an observationally based explanation why NO_x_ concentrations have hardly declined since the introduction of EURO 3 emission standards in Central Europe. While the technological shift towards Diesel passenger cars might have helped curb CO_2_ emissions through better fuel economy compared to gasoline powered cars in the past, it created a widespread problem of NO_2_ pollution across Europe^23^ that does not seem to be exclusively limited to the largest metropolitan and industrialized areas. The presented flux measurements indicate that traffic related NO_x_ emissions in current operational air quality models can be significantly underestimated by up to a factor of 4 across countries exhibiting a sizeable fraction of Diesel powered cars in their fleet. As Diesel fuels (including bio-diesel) could account for 70% of the growth in transportation fuels by 2040, with significant demand in Asian markets according to industry projections^57^, a better understanding of the uncertainty in associated changes of NO_x_ fluxes and ozone chemistry will therefore be important for future environmental impact studies. Our measurements show that projected significant decreases in European NO_x_ emissions from the mobile transport sector will lead to conditions improving NO_2_ exposure limits, but could locally increase ozone levels on the short term. Concomitant reduction measures for NO_x_ and NMVOC (CO) might therefore still prove most effective to avoid parallel increases of local ozone levels due to new NO_x_ emission standards. This might be particularly important in areas where topographic amplification can lead to a stronger accumulation of air pollutants than over flat land and be a relevant consideration for mountainous mega-cities^58^. Using the observed weekend effect as proxy for underestimated NO_x_ emissions (i.e. a factor 2–4 difference), models would overestimate P(O_3_) by 30–40% under the observed NO_x_ inhibited – VOC limited regime and underestimate P(O_3_) downwind, once NO_x_ concentrations fall below 2–4 ppbv. Here we demonstrate that parallel flux measurements of a wide range of chemical species can be used to benchmark urban emission sources, complement traditional approaches and significantly improve uncertainties inherent to bottom-up scaling in atmospheric chemistry models.

## Methods

### Instrumentation

#### NMVOC

A PTR-QiTOF instrument (Ionicon, Austria) was operated in hydronium mode at standard conditions in the drift tube of 112 Townsend. The instrument was set up to sample ambient air from a turbulently purged “3/8” Teflon line. Every seven hours, zero calibrations were performed for 30 minutes providing VOC free air from a continuously purged catalytical converter though a setup of software controlled solenoid valves. In addition, known quantities of a suite of VOC from a 1 ppm calibration gas standard (Apel & Riemer, USA) were periodically added to the VOC free air and dynamically diluted into low ppbv mixing ratios. ***NO***_***x***_ ***:*** A dual channel chemiluminescence instrument (CLD 899 Y; Ecophysics) was used for NO and NO_x_ measurements. The instrument was equipped with a metal oxide converter operated at 375C. The instrument was operated in flux mode acquiring data at about 5 Hz, similar to measurements performed over a pasture^59^ and forest^60^. A NO standard was periodically introduced for calibration. Zeroing was performed once a day close to midnight. ***CO***_***2***_, ***H***_***2***_ ***O***: A closed path eddy covariance system (CPEC 200; short inlet, enclosed IRGA design; Campbell Scientific) measured three dimensional winds along with CO_2_ and H_2_O. An additional 3D sonic anemometer (CSAT3; Campbell Scientific) was available for turbulence measurements at an alternative height level (Fig. S3). Calibration for CO_2_ was performed once a day. ***O***_***3***_ ***:*** Ozone concentrations were obtained from a closed-path UV photometric analyser (APOA-370, Horiba, Japan); ***CO***: CO measurements were available for a limited amount of time in August 2015. Ambient mole fractions of carbon monoxide (CO) were measured with a quantum cascade laser spectrometer (CWQC-TILDAS-76-D, Aerodyne, USA) with a 76 m path length optical cell at a wavenumber of ca. 2190 cm^−1^. The QCL was operated at a pressure of ca. 4 kPa using a built-in pressure controller and temperature of the optical bench and housing controlled to 35 °C. Fitting of absorption spectra at 2 Hz, storing of calculated dry mole fractions, switching of zero/calibration valves, control of pressure lock, correction for band broadening and other system controls were realized by the TDLWintel software (Aerodyne, USA) run on a PC synchronized in time with the system collecting the anemometer data using the NTP software (Meinberg, Germany).

#### Eddy covariance data analysis

The eddy covariance method is derived from the scalar budget equation after Reynolds decomposition, and in its simplest form for horizontally homogeneous flows normal to the surface, where the mean vertical motion of wind ($\bar{w}$) can be considered 0, relates the measured surface-atmosphere exchange flux (F) to the covariance between vertical wind and concentration fluctuation according to:

$${\rm{F}}=\langle w^{\prime} c^{\prime} \rangle ,$$

where w′ represents the vertical fluctuation of wind speed, and c′ the concentration fluctuation. Brackets denote the averaging interval. The ensemble average used here is 30 minutes. Fluxes were selected according to standard quality control criteria, such as raw data despiking, correcting for high and low pass filtering biases, applying a stationarity test and test on developed turbulent conditions (e.g. u* filtering)^61^. In addition we parsed the data to make sure that the flux footprint would reflect a representative urban area (*SI*).

## Electronic supplementary material

Supplementary Info
