# Supplementary material for: Urban eddy covariance measurements reveal significant missing NOx emissions in Central Europe
Source: Sci Rep. 2017 May 30;7:2536. doi: 10.1038/s41598-017-02699-9 (PMC5449400; doi:10.1038/s41598-017-02699-9)
Supplement: Supplementary file 1 — Supplementary Info [file 41598_2017_2699_MOESM1_ESM.pdf]

Supplementary Information

Urban eddy covariance measurements reveal significant missing NO_x emissions in Central Europe

Authors: T. Karl[1*], M. Graus[1], M. Striednig[1], C. Lamprecht[1], A. Hammerle[2], G. Wohlfahrt[2], A. Held[3], L. von der Heyden[3], M.J. Deventer[4], A. Krismer[5], C. Haun[6], R. Feichter[7], J. Lee[8]

Affiliations:

[1] Institute of Atmospheric and Cryospheric Sciences, University of Innsbruck, Austria.

[2] Institute of Ecology, University of Innsbruck, Austria.

[3] Atmospheric Chemistry, University of Bayreuth, Germany.

[4] Department of Geography, University of California, Berkeley, USA.

[5] Abteilung Waldschutz, Amt der Tiroler Landesregierung, Austria.

[6] Abteilung Geoinformation, Amt der Tiroler Landesregierung, Austria.

[7] Amt für Verkehrsplanung, Umwelt, Magistrat III Stadt Innsbruck, Austria

[8] National Centre for Atmospheric Science and Department of Chemistry, University of York, U.K.

*Correspondence to: thomas.karl@uibk.ac.at

Supplementary Information:

Field Site and Local Meteorology

The iNNAQS (iNNsbruck Air Quality) study took place between 1. July 2015 – 30. October 2015. Fig. S1 shows the sampling location (black dot) situated close to the city center of Innsbruck (Lat: 47°15'51.66" Lon:11°23'06.82"). The regions exceeds air quality standards for NO_2 regularly (Fig. S2).

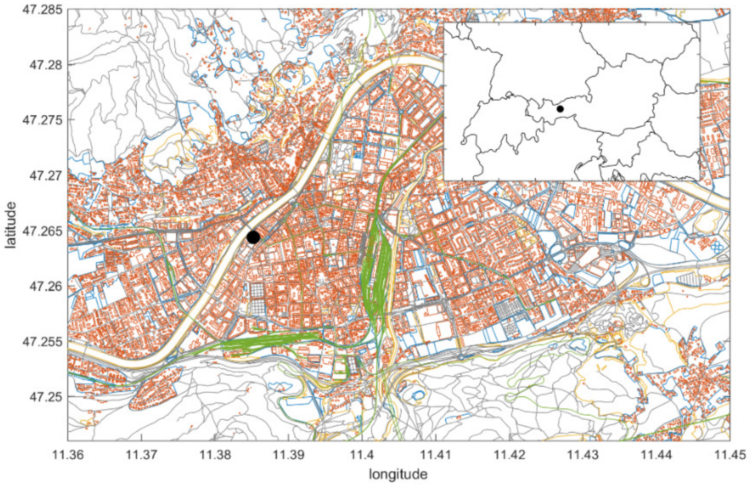

Figure S1. Measurement site (black dot). Maps were created in Matlab (R2015b version 8.6.0.267246, Mathworks, USA, https://de.mathworks.com/) based on OpenStreetMap (https://www.openstreetmap.org/copyright).

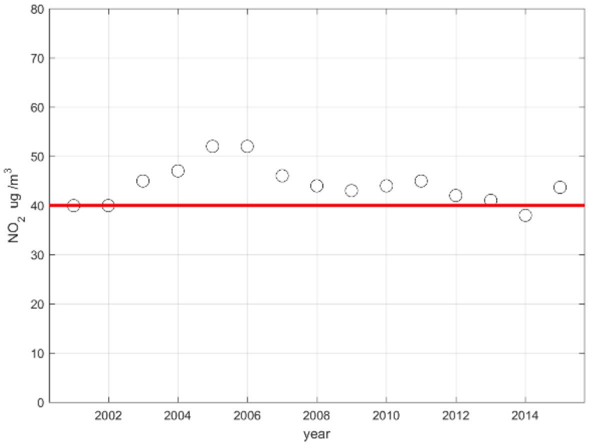

Figure S2: Evolution of yearly averaged NO_2 concentrations at an air quality station (11° 23' 32.5", 47° 15' 45.4") within ~700 m distance of the INNAQS flux tower. Red line indicates the regulatory threshold. Euro 3 standards were introduced in January 2000.

Inlets for turbulent flux measurements were situated at a tower on top of the Bruno-Sander building 38.6 m above street level and about twice the average building height within the NE to SE fetch. Fig. S3 depicts vertical profiles of friction velocity in comparison with data obtained in Zürich (*Rotach, 1993*) suggesting that the iNNAQS tower is situated close to the top of the roughness sublayer where flux profiles become constant.

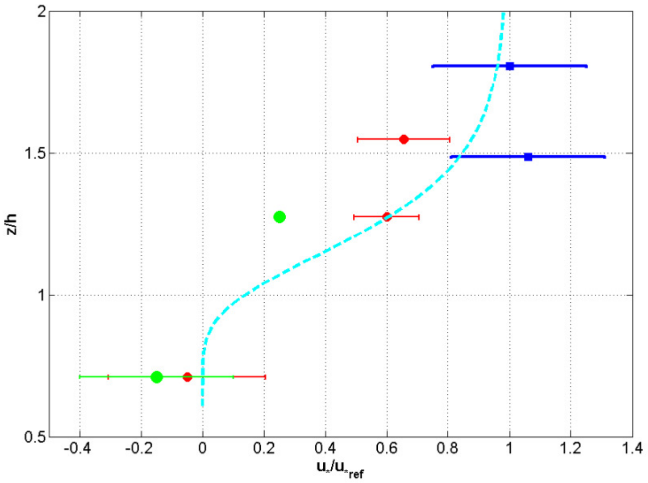

Figure S3: Relative friction velocity vs. normalized height. Blue symbols are obtained from this work, green (red) symbols are adopted Zürich (Rotach, 1993) measured at the face of the building (green) and in the middle of the street canyon (red). The light blue line is a fitted parametric model.

The general wind direction (Fig. S4) followed the expected valley wind system where most of the flux data for sensible heat, trace gases and aerosols are captured along the NE (~60◦ ~33 % of the time) and SW (~220◦ ~41% of the time) valley axis. The NE (40◦-90◦) corridor captures most of the inner-city of Innsbruck and is representative of a typical urban fingerprint. The SW sector (160◦-260◦) represents mostly an urban residential area. 70% and 51% of sensible heat and CO_2 fluxes passed the stationarity test (Foken et al., 2010).

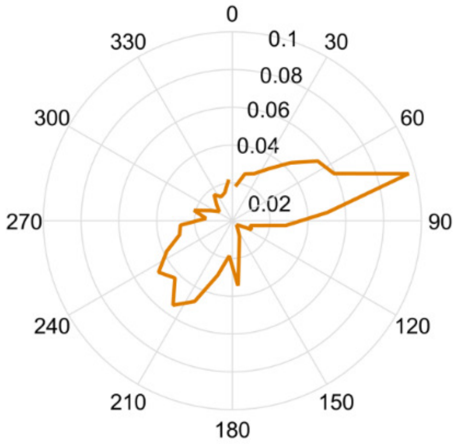

Figure S4: Relative Distribution of the general wind direction at the site.

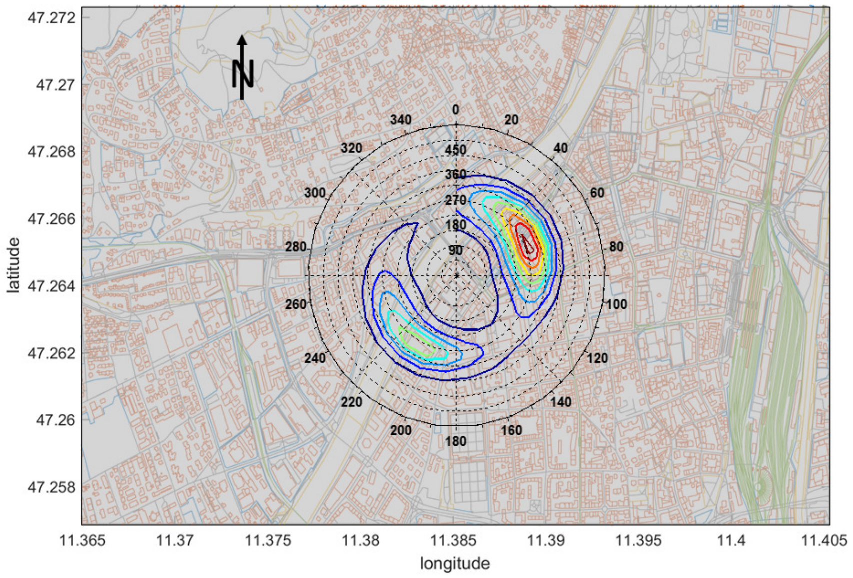

Figure S5: 80% Flux footprint contours (peaks at ~300 -320m). Distance plotted vs wind direction (Kljun et al., 2015). Maps were created in Matlab (R2015b version 8.6.0.267246, Mathworks, USA, https://de.mathworks.com/) based on OpenStreetMap (https://www.openstreetmap.org/copyright).

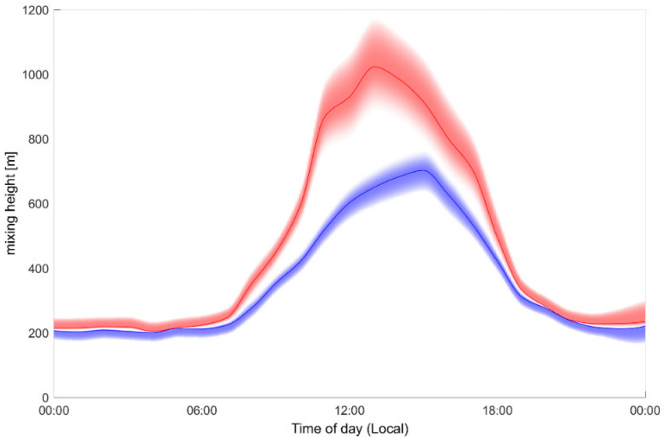

Figure S6: Calculated upper and lower range of mixing layer height during INNAQS estimated based on a passive microwave sounding (Massaro et al., 2015) according to two parcel methods (lower range – simple parcel method; upper range – advanced parcel method, Seibert et al., 2000). Solid lines depict the average diurnal profiles and the shading shows the respective 95% confidence interval.

Eddy covariance flux measurements

Eddy covariance fluxes were calculated as the covariance between the rotated vertical wind speed and the tracer mole fraction using routines described previously (**http://www.geos.ed.ac.uk/homes/rclement/micromet/EdiRe/**, Karl et al., 2002). Quality control was performed according to procedures described by Foken et al. (2010). Systematic errors due to high frequency losses were obtained from co-spectral analysis according to Massman (2010) and are summarized in the table below:

Table S1: High frequency damping and fractional loss of the missing high frequency contribution, determined experimentally and calculated according to a parameterization (Massmann, 2010)

	CO_2	H_2O	NMVOC	NO, NO_x
Damping timescale	0.2 s	0.7 s	0.4 s	0.8 s
Loss	4%	12%	7%	13%

Inventories and Models

Traffic simulation models:

The COPERT (v4) software package allows calculating air pollutant and greenhouse gas emissions from road transport. The development of COPERT is coordinated by the European Environment Agency (EEA), in the framework of the activities of the European Topic Centre for Air Pollution and Climate Change Mitigation. COPERT has been developed for official road transport emission inventory preparation in EEA member countries (http://emisia.com/products/copert-4). Here we initialized the model with Austrian fleet statistics obtained from the TRACCS (Transport data collection supporting the quantitative analysis of measures relating to transport and climate change) database (*33*) with data for 2010. A correction factor of 1.2 was applied to calculated COPERT NO_x emission factors taking into account reported emission changes between the year 2010 and 2015 according to the HBFA 3.2 database retrieved on 1.3.2016: http://www.hbefa.net/d A similar factor was found when adjusting for the GAINS (Greenhouse Gas - Air Pollution Interactions and Synergies Model: http://gains.iiasa.ac.at/models/) (23) trend between 2010 and 2015.

The Atmospheric Chemistry and Climate Model Intercomparison Project (ACCMIP) and EMEP regional emission inventory:

ACCMIP emission datasets were retrieved on April 1st 2016 as gridded (0.1 degree) netcdf files for 2015 from the ACCMIP data server http://accmip-emis.iek.fz-juelich.de/data/accmip/gridded_netcdf/ via http://www.geiacenter.org/. A detailed description about ACCMIP can be found as part of a special issue http://www.atmos-chem-phys.net/special_issue296.html. (*48*). Gridded data were extracted for Central Europe as depicted in Fig. S7. We applied the experimentally determined CO/CO_2 emission ratio to INNAQS data for comparison with ACCMIP.

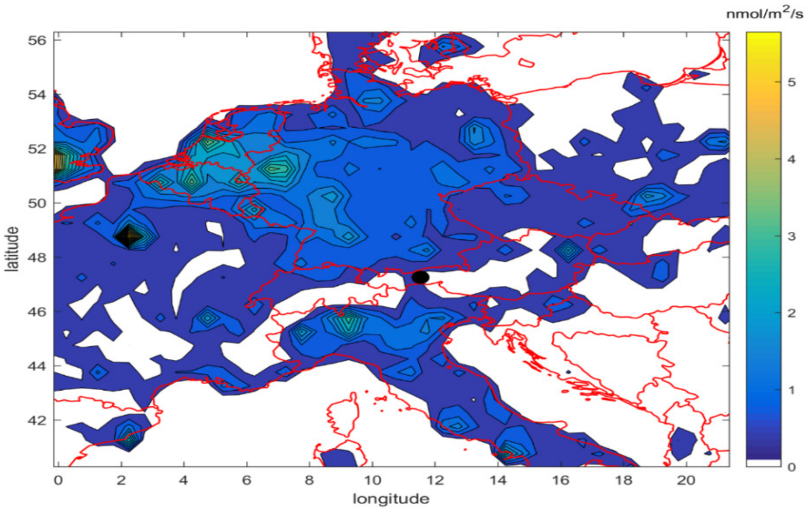

Figure S7: Average (June-October 2015) gridded NO_x emissions for domestic and traffic related sources plotted with Matlab (R2015b version 8.6.0.267246, Mathworks, USA, https://de.mathworks.com/). The black dot shows the

measurement site. For better clarity the color bar is truncated below 0.2 and above 6.0 nmol/m^2/s. NO_x data were obtained via http://accmip-emis.iek.fz-juelich.de/data/accmip/gridded_netcdf/.

EMEP (European Monitoring and Evaluation Program) emission data were retrieved from the center on emission inventories and projections via the ECCAD (Emissions of atmospheric compounds & compilation of Ancillary Data) project (http://eccad.sedoo.fr/eccad_extract_interface/JSF/page_login.jsf) on August 1st 2016. The inventory represents officially reported national data mandated by the Convention on long range transboundary air pollution treaty http://www.ceip.at/ms/ceip_home1/ceip_home/webdab_emepdatabase/reported_emissiondata/).

Emission ratio model

The Matlab (R2015b, Mathworks, USA) nonlinear programming solver "fmincon" was used to fit a constrained multivariable model to obtain the two end-members representing urban residential combustion and traffic specific emission ratios (e.g NO_x/CO_2):

$a_{x1}r_1 + a_{x2}r_2 = r_{mx}$, where a_{x1} and a_{x2}, represent the activity of the two sources (i.e. urban residential combustion and traffic), r_1 and r_2 are the emission ratios for sources 1 and 2 and r_{mx} is the combined measured ratio for each time step x. The activity factors for each time step x are subject to $a_{x1} + a_{x2} = 1$, with x=1:24.

Traffic count data and stationary emission inventory

Traffic count data were provided by the local transportation department, which maintains 18 traffic count stations within the city of Innsbruck. For the analysis here, we used data from Innrain 51, which is situated next to the measurement site, and represents the activity along the major E-W traffic route within the flux footprint. Since our analysis only requires relative changes, we used Innrain 51 as a proxy, but note that all traffic stations show a very similar relative weekend to weekday and diurnal behavior. Bounds for emission ratios for stationary sources were obtained from reported emission factors for residential heating systems according to the CORINAIR /SNAP classification (1.1., 1.2., 2.2., 2.2, 8.9), available for the local legislative district. Emission factors applied to stationary sources are based on a detailed Austrian assessment (Haun, 2010; Stanzel et al., 1995)

The Leeds Master Chemical Mechanism (MCMv3.2).

The Master Chemical Mechanism developed by the National Centre for Atmospheric Sciences at the University of Leeds summarizes the state of art knowledge on tropospheric chemistry. The chemical mechanistic information was taken from the Master Chemical Mechanism, MCM v3.2 (*13*) via the website: http://mcm.leeds.ac.uk/MCM and processed for further analysis in Matlab (www.mathworks.com) using the box model version of CAFÉ (*55*). We setup the model in two modes.

Fully constrained setup:

The full MCM model was initiated with measurements according to Table S2 and run for 10 minutes to let radicals evolve to quasi steady state (*19*). O_3, NO_x and NMVOC were changed according to the weekend effect. A sensitivity study was performed to change NO_x concentrations over a wide range. The gross ozone production is calculated as $P(O_3)$=k(NO x HO_2 + NO x RO_2. The total reactivity (without NO_x) at 10 ppbv NOx was between 2.7 to 6 s^{-1} in these runs (initial reactivity of about 2.7 to 3 s^{-1}).

Diurnally constrained setup:

For these runs the full MCM was setup as a diurnally varying constrained model framework as was previously described in similar types of studies (e.g. Crawford et al., 1999; Frost et al., 2002). The model was fully constrained by all measured species, except NO_2 and O_3, which were only initialized once and then allowed to be continuously updated by corresponding model values. Species listed in Table S2 were constrained by actual measurements during each model integration step and followed the observed diurnal cycle. Photolysis rates were incorporated from measurements of shortwave radiation according to Trebs et al. (2009). Meteorological data (temperature, humidity) were taken from measurements. Time integration of the chemical rate equations was started at midnight and performed for 3 days to let the model spin up. The height of the planetary boundary layer /mixing height (PBL) was taken from measurements (Fig. S6). We take the average of the upper (red) and lower (blue) limit for PBL estimations. The overall dilution of the model was incorporated according to:

$$\frac{d[C_{PBL}]}{dt}_{entrainment} = -K_{entrainment}\left([C]_{PBL} - [C]_{bg}\right) \text{ eq. S1,}$$

where $[C_{PBL}]$ corresponds to the PBL concentration, $[C_{bg}]$is the background concentration, and $K_{entrainment}$ summarizes all transport/dilution losses. The first order rate constant ($K_{entrainment}$) was calculated from the measured PBL growth, and a constant dilution factor proportional to $1.6e^{-5}$ s^{-1} which incorporates effects of mountain-valley circulation happening on a typical timescale of 8h. Ozone deposition was assumed to be negligible compared to these transport terms. Background concentrations for O_3 were taken as 30 ppbv, and 10% of PBL NO_2, corresponding to typical background conditions obtained from mountain top stations. All other measured species listed in Table S2 were constrained by diurnal observations. Concentrations for photo-chemically produced species that could not be constrained by measurements were initially set to 0.

Table S2: Typical MCM model setup for a fully constrained model run. All MCM species that are not listed were not constrained by initial conditions. To simulate radical production on weekends the initial concentrations for NMVOC were downscaled proportional to benzene or observations were available. IC=initial condition.

Variable	Data range	Notes
P (mbar)	1013	Constrained to IC
T (K)	298	Constrained to IC
RH (%)	60	Constrained to IC
Solar zenith angle (°)	33	Constrained to IC
**MCM species in ppbv**		
NO	.01 to 6	Split into 25 intervals (not constrained)
NO_2	.1 to 30	Split into 25 intervals (constrained to IC)
O_3	50, 100, 150	3 scenarios (not constrained)
CH_4	1150	(constrained to IC)
CO	100	measured (constrained to IC)
'BENZENE'	0.1	measured (constrained to IC)
'TOLUENE'	0.25	measured (constrained to IC)
'OXYL'	0.05	measured and scaled (constrained to IC)
'MXYL'	0.07	measured and scaled (constrained to IC)
'PXYL'	0.07	measured and scaled (constrained to IC)
'EBENZ'	0.045	measured and scaled (constrained to IC)
'C2H4'	0.875	scaled to benzene (constrained to IC)
'C2H6'	1.43	scaled to benzene (constrained to IC)
'C3H6'	0.32	scaled to benzene (constrained to IC)
'C3H8'	0.955	scaled to benzene (constrained to IC)
'NC4H10'	0.91	Sum of all butanes scaled to benzene (constrained to IC)

'BUT1ENE'	0.035	Sum of all butenes scaled to benzene (constrained to IC)
'C2H2'	0.5	scaled to benzene (constrained to IC)
'CH3COCH3'	1.125	measured (constrained to IC)
'C5H8'	0.2	measured (constrained to IC)
'APINENE'	0.1	measured and evenly split between the two MTs in the MCM (constrained to IC)
'BPINENE'	0.1	measured and evenly split between the two MTs in the MCM (constrained to IC)
'CH3OH'	2	measured (constrained to IC)
'HCHO'	1	a priori model assumption (not constrained)
'CH3CHO'	0.47	measured (not constrained)
'C4H6'	0.025	scaled to benzene (constrained to IC)

Supplemental references:

Crawford, J., Davis, D., Olson, J., Chen, G., Liu, s., Gregory, G., Barrick, J., Sachse, G., Sandholm, S., Heikes, B., Singh, H., & D. Blake, Assessment of upper tropospheric HOx sources over the tropical Pacific based on NASA GTE/PEM data: Net effect on HOx and other photochemical parameters, *J. Geophys. Res.,* **107**, doi: 10.1029/1999JD900106, (1999).

Foken, T., Leuning, R., Oncley, S.P., Mauder, M. & M. Aubinet, *Corrections and data quality, Eddy Covariance: A Practical Guide to Measurement and Data Analysis* (Kluwer Academic Publishers, Dortrecht, ed. 1, 2010), pp. 85-131.

Frost, G.J., Fried, A., Lee, Y.N., Wert, B., Henry, B., Drummond, J.R., Evans, M.J., Fehsenfeld, F.C., Goldan, P.D., Holloway, J.S., Hübler, G., Jakoubek, ., Jobson, B.T., Knapp, K., Kuster, W.C., Roberts, J., Rudolph, J., Ryerson, T.B., Stohl, A., Stroud, C., Sueper, D.T., Trainer, M., & J. Williams, Comparison of box model calculations and measurements of formaldehyde from the 1997 North Atlantic regional Experiment, *J. Geophys. Res.,* **107**, doi: 10.1029/2001JD000896 (2002).

Haun, C., „Emissionskataster Tirol“ (Tech. Report., Amt der Tiroler Landesregierung, https://www.tirol.gv.at/fileadmin/themen/sicherheit/geoinformation/Geoinformation_EMIKAT_Hausbrand_2010/EMIKATHausbrandBerichtkomplett.pdf, 2010) [the easiest access to this source is via the URL].

Karl, T., Spirig, C., Rinne, J., Stroud, C., Prevost, P., Greenberg, J., Fall, R. & A. Guenther, Virtual disjunct eddy covariance measurements of organic compound fluxes from a subalpine forest using proton transfer reaction mass spectrometry, *Atmos. Chem. Phys,* **2**, 279-291 (2002).

Kljun, N., Calanca, P., Rotach, M.W., & H.P. Schmid, A simple two-dimensional parameterization for flux footprint prediction (FFP), *Geosci. Model Dev.*, **8**, 369-3713 (2015)

Massaro, G., Stiperski, I., Pospichal, B., & M.W. Rotach, Accuracy of retrieving temperature and humidity profiles by ground-based microwave radiometry in truly complex terrain, *Atmos. Meas. Tech*., **8**, 3355-3367 (2015)

Massmann, W., & R. Clement, *Uncertainty in Eddy Covariance Flux Estimates Resulting from Spectral Attenuation: A Practical Guide to Measurement and Data Analysis* (Kluwer Academic Publishers, Dortrecht, ed. 1, 2010), pp. 67-96.

Rotach, M.W., Turbulence close to a rough urban surface 1. Reynolds stress, *Bound. Lay. Meteo.*, 65, 1-28 (1993).

Seibert, P., Beyrich, F., Gryning, S.E., Joffre, S., Rasmussen, A., & P. Tercier, Review and intercomparison of operational methods for the determination of the mixing height. *Atmospheric Environment*, 2000. **34**, 1001-1027, DOI: http://dx.doi.org/10.1016/S1352-2310(99)00349-0. (2000)

Stanzel, W., Jungmeier, G., & J. Spitzer „Emissionsfaktoren und energietechnische Parameter für die Erstellung von Energie- und Emissionsbilanzen im Bereich Raumwärmeversorgung“ (Tech. Report IEF-B-02/95, Joanneum Research, Graz, 1995).

Trebs, I., Bohn, b., Ammann, C., Rummel, U., Blumthaler, M., Königstedt, R., Meixner, F.X., Fan, S., & M.O. Andreae, *Atmos. Meas. Tech*., **2**, 725-739, (2009)
